# Fibrous Hydrogels for Cell Encapsulation: A Modular and Supramolecular Approach

**DOI:** 10.1371/journal.pone.0155625

**Published:** 2016-05-25

**Authors:** Małgorzata K. Włodarczyk-Biegun, Kambiz Farbod, Marc W. T. Werten, Cornelis J. Slingerland, Frits A. de Wolf, Jeroen J. J. P. van den Beucken, Sander C. G. Leeuwenburgh, Martien A. Cohen Stuart, Marleen Kamperman

**Affiliations:** 1 Physical Chemistry and Soft Matter, Wageningen University & Research, Wageningen, The Netherlands; 2 Department of Biomaterials, Radboud University Medical Center, Nijmegen, The Netherlands; 3 Wageningen UR Food & Biobased Research, Wageningen, The Netherlands; University of California, San Diego, UNITED STATES

## Abstract

Artificial 3-dimensional (3D) cell culture systems, which mimic the extracellular matrix (ECM), hold great potential as models to study cellular processes under controlled conditions. The natural ECM is a 3D structure composed of a fibrous hydrogel that provides both mechanical and biochemical cues to instruct cell behavior. Here we present an ECM-mimicking genetically engineered protein-based hydrogel as a 3D cell culture system that combines several key features: (1) Mild and straightforward encapsulation meters (1) ease of ut I am not so sure.encapsulation of the cells, without the need of an external crosslinker. (2) Supramolecular assembly resulting in a fibrous architecture that recapitulates some of the unique mechanical characteristics of the ECM, i.e. strain-stiffening and self-healing behavior. (3) A modular approach allowing controlled incorporation of the biochemical cue density (integrin binding RGD domains). We tested the gels by encapsulating MG-63 osteoblastic cells and found that encapsulated cells not only respond to higher RGD density, but also to overall gel concentration. Cells in 1% and 2% (weight fraction) protein gels showed spreading and proliferation, provided a relative RGD density of at least 50%. In contrast, in 4% gels very little spreading and proliferation occurred, even for a relative RGD density of 100%. The independent control over both mechanical and biochemical cues obtained in this modular approach renders our hydrogels suitable to study cellular responses under highly defined conditions.

## Introduction

In natural tissues, most cells interact with the native extracellular matrix (ECM) in a 3-dimensional (3D) environment [1,2]. The ECM, a fibrous mesh of high complexity and hierarchy, ensures proper molecular structure, functional bioactivity, and mechanical support for cells [2]. Mutual cell–ECM interactions form a dynamic regulatory system, directing cell behavior [1] and thereby influencing tissue formation and regeneration [3].

Current knowledge about cell–matrix interactions is mostly based on 2-dimensional (2D) *in vitro* studies. However, culturing cells in a monolayer does not accurately represent the conditions in living tissues and affects several important aspects, such as cell adhesion and functionality, the biomechanics of the system, and interactions of cells with solutes [4]. Not surprisingly, several studies have shown considerable differences between cellular responses in 2D and 3D [4,5,6,7]. Motivated by the suggestion that 3D systems might bridge the gap between traditional 2D culture and animal models [8,9], researchers have been developing ECM-mimetic 3D cell culture matrices from several material classes. Materials derived from natural sources usually ensure high biocompatibility and the presence of bioactive domains. However, they may reveal batch-to-batch variations and can be contaminated with disease agents. Moreover, precise control over properties is not possible [2,4,9,10]. Chemically synthesized materials have also been used as 3D cell culture matrices and offer much more control [2,4,9], although biocompatibility can be a limiting factor [2,10] and precision is still restricted. An interesting alternative is provided by protein-based polymers. These are produced biotechnologically as recombinant proteins encoded by synthetic genes, which allows customization of the design by precise control over amino acid sequence and molecular weight. Protein-based polymer materials are generally monodisperse and functionalization of scaffolds is possible through introduction of genetically encoded bioactive sites [11]. Several 3D protein- based polymer hydrogel matrices for cell culture have been reported [1,11,12,13,14,15,16,17]. These studies have identified key factors for the suitability of hydrogels as 3D scaffolds: (1) mild encapsulation conditions for the cells [1,17], (2) biomechanical features of the gels, such as a fibrous architecture and resulting matrix stiffness and yield stress [1,6,18], and (3) introduction of biochemical signals, such as cell-adhesive motifs [1,12].

**Fig 1:**
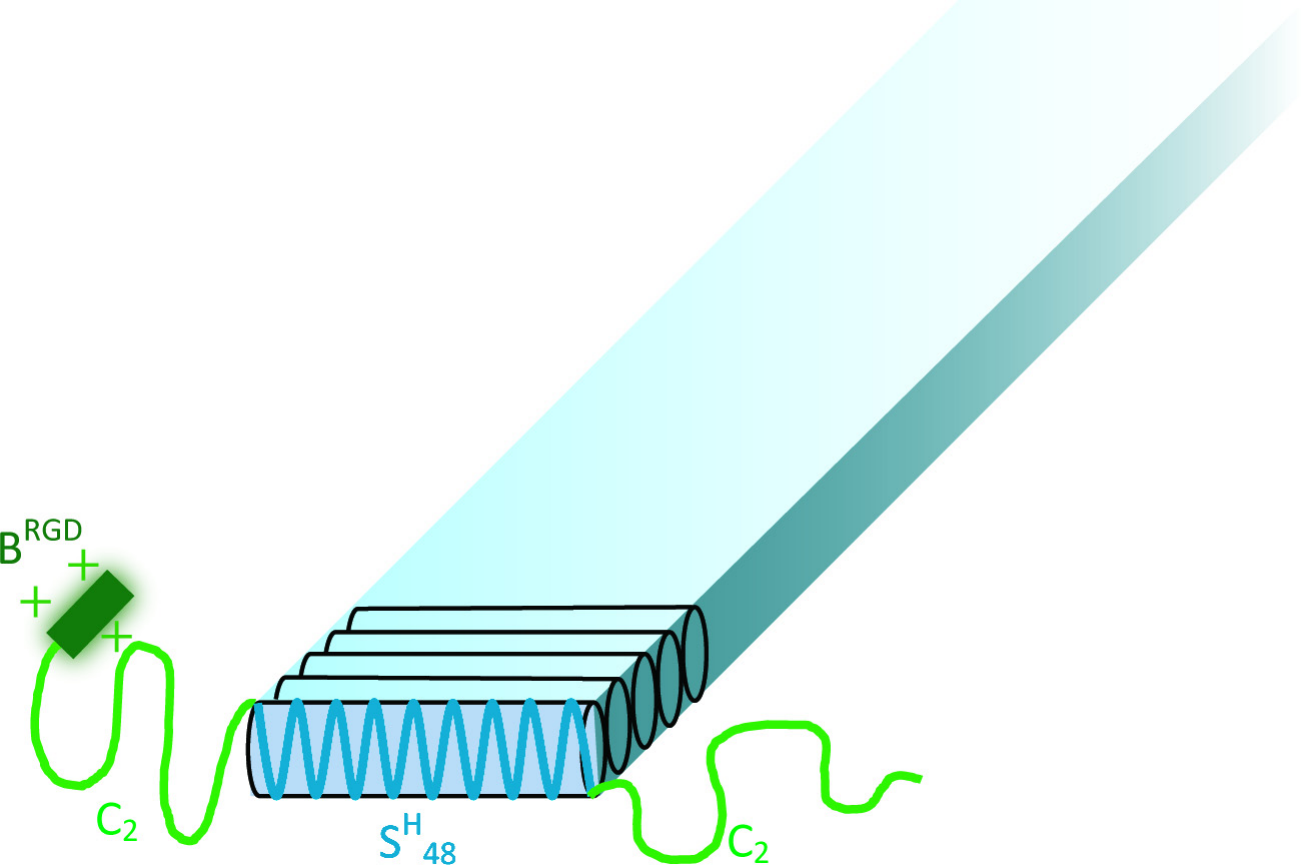
Tentative structure of a B^RGD^C_2_S^H^_48_C_2_ fiber at neutral pH. Drawing not to scale. **B**^**RGD**^ consists of 24 residues, and the entire protein sequence is 826 residues.

The aim of this study is to investigate an ECM-mimicking genetically engineered protein-based hydrogel system that combines the abovementioned three key factors, as a new material for 3D cell culture scaffolds. The modular approach we use allows for mutually independent control over material properties, i.e., the RGD domain density and hydrogel concentration. In this way, we analyze which material parameters significantly influence behavior of encapsulated cells. Our system’s basis is a silk-inspired protein-based triblock copolymer, further denoted as **C**_**2**_**S**^**H**^_**48**_**C**_**2**_ [19]. It consists of a **s**ilk-like, histidine-containing (GAGAGAGH)_48_ middle block, further denoted as **S**^**H**^_**48**_, flanked on both sides by hydrophilic random **c**oil end blocks, further denoted as **C**_**2**_ (see Fig 1). The **S**^**H**^_**48**_ block assumes a β-roll conformation and drives fiber formation upon pH-triggered neutralization of the positively charged histidines [20,21]. Each of the **C**_**2**_ blocks consists of two hydrophilic, 99 amino acid-long domains in tandem, which form random coils regardless of pH and provide colloidal stability to the fibers. Thus, this polymer system is soluble at low pH and self-assembles into supramolecular fibers and hydrogels at physiological pH. This allows mild and straightforward encapsulation of the cells, by adding cells to the protein solution at an early stage of gel formation at physiological pH, without the need of an external crosslinker. The fibrous and supramolecular nature of the gel recapitulates some of the unique mechanical characteristics of the ECM, i.e. strain-stiffening and self-healing behaviour. In addition, the stiffness of the matrix can be tuned by changing the protein polymer concentration.

In this study we co-assemble **C**_**2**_**S**^**H**^_**48**_**C**_**2**_ with a cell-adhesive variant. This variant is denoted as **B**^**RGD**^**C**_**2**_**S**^**H**^_**48**_**C**_**2**_, which contains at its N-terminus two, well-known, cell-**b**inding GRGDSP motifs separated by a flexible (GGSG)_3_ spacer. By mixing protein-based polymers with and without RGD domains in any desired ratio, the biochemical cue density is precisely and easily controlled. We have assessed the performance of this modular system as a matrix for MG-63 osteoblastic cells. This cell line was selected, because the integrin subunits profile of MG-63 is very similar to the human profile [22], which is an important factor in our study. To identify possible limitations and advantages of our material system we investigated the effect of two variables: RGD domain density and hydrogel concentration.

## Materials and Methods

### Protein-based polymers

The design and production in *P*. *pastoris* of the genetically engineered protein-based polymers **C**_**2**_**S**^**H**^_**48**_**C**_**2**_ and **B**^**RGD**^**C**_**2**_**S**^**H**^_**48**_**C**_**2**_ was described recently by us [19,23,24].

### Protein sample preparation

Throughout the study, samples with three different adhesive motif densities and three different total protein concentrations were used. For clarity every sample is assigned a unique code. Samples consisting of either (a) **C**_**2**_**S**^**H**^_**48**_**C**_**2**_, or (b) **B**^**RGD**^**C**_**2**_**S**^**H**^_**48**_**C**_**2**_, or (c) **C**_**2**_**S**^**H**^_**48**_**C**_**2**_ mixed with **B**^**RGD**^**C**_**2**_**S**^**H**^_**48**_**C**_**2**_ in a 1:1 weight ratio are denoted as (a) 0B (no binding domains), (b) 100B (100% protein with binding domains), and (c) 50B (50% protein with binding domains and 50% unmodified protein), respectively. The sample codes subsequently indicate the adhesive motif density and final protein concentrations of 1%, 2%, or 4% (w/v). An overview of all samples and the assigned codes is given in Table 1.

**Table 1:** Sample codes, relative adhesive motifs density, and total protein concentration.

Protein sample code	Relative RGD density (percentage of functionalized protein)		Protein final concentration (w/v)
	C_2_S^H^_48_C_2_	B^RGD^C_2_S^H^_48_C_2_	
0B 1%	100%	0%	1%
0B 2%	100%	0%	2%
0B 4%	100%	0%	4%
50B 1%	50%	50%	1%
50B 2%	50%	50%	2%
50B 2% WP*	50%	50%	2%
50B 2% NB*	50%	50%	2%
50B 4%	50%	50%	4%
100B 1%	0%	100%	1%
100B 2%	0%	100%	2%
100B 4%	0%	100%	4%

*WP = well-plate, NB = no HEPES buffer.

To prepare the samples, freeze-dried proteins were dissolved in 10 mM HCl (Merck), at a 2-fold protein concentration compared to that in the final sample, and vortexed with a multi-tube holder for 1.5 hours. For the 50B sample, **C**_**2**_**S**^**H**^_**48**_**C**_**2**_ and **B**^**RGD**^**C**_**2**_**S**^**H**^_**48**_**C**_**2**_ were mixed after dissolving at low pH. Next, final protein concentrations and pH were adjusted with 0.1 M NaOH (Merck), 10 x concentrated phosphate buffered saline (PBS, pH 7.4, Lonza) and HEPES buffer (Fisher Scientific). Final solutions contained 1 x concentrated PBS and 25 mM HEPES. Additionally, two variants of 50B 2% were prepared as controls: (1) To estimate the influence of well-plate design on nutrient supply in cell cultures, a sample was cast directly on the bottom of a 96-well plate, instead of a cell culture insert in 24-well plates as performed usually (see section 2.5). The pH was adjusted as described above. This sample is further denoted as 50B 2% WP (96-well plate). (2) To exclude possible effects of transient high buffer and salt concentration during sample preparation, a sample was prepared at the final protein concentration, where the pH was adjusted by addition of 0.1 M NaOH and 1 x concentrated PBS (instead of 10 x concentrated), without the addition of HEPES buffer. This sample is further denoted as 50B 2% NB (no HEPES buffer).

### Diffusion study

Since pore size could be an important parameter for cell viability, migration, and spreading, diffusion of dextran molecules of various sizes was used to estimate the pore size of the gels with different protein concentrations. Samples 0B 1%, 0B 2%, 0B 4%, 100B 1%, 100B 2%, and 100B 4% of 250 μl were transferred immediately after preparation to 1.5 ml glass vials, subsequently closed with a cap to avoid evaporation, and left at room temperature to allow gelation. After 48 hours, 500 μl solutions containing fluorescein isothiocyanate (FITC)-labelled dextrans of different average molecular weights (20, 70, and 500 kDa, Sigma), were deposited on top of each gel. Based on a study by Armstrong et al. [25], the hydrodynamic radii of the particles in these aqueous solutions were determined as 3.3, 6.5, and 15.9 nm, respectively. Dextrans were dissolved at 200 μg/ml in a mixture of PBS, HEPES (25 mM) and sodium azide (0.001% (w/v)). All samples were prepared in triplicate (n = 3). After 11 days of incubation, the concentration of dextran in the gels was determined by measuring the FITC fluorescence (excitation: 485 nm; emission: 535 nm) with a SpectraMax M2 Microplate Reader (Molecular Devices). To this end, gels were dissolved in 10 mM HCl prior to the measurements. The partition coefficient was defined as:

$$Partition\;coefficient=\frac{C_{gel}}{C_{liquid}}$$ (1)

*C*_*gel*_ is the concentration of FITC-dextran determined in the gel, corrected for the volume of added HCl, and *C*_*liquid*_ is the concentration of FITC-dextran remaining in the liquid layer on top of the gel.

### Rheological characterization of hydrogels

0B, 50B, and 100B samples were tested at three different total protein concentrations (1%, 2%, and 4% (w/v)) at pH 7.4 ± 0.2 and 37°C. Immediately after preparation, protein solutions were loaded into the rheometer (Physica MCR 501 Rheometer, Anton Paar) equipped with a Couette CC10/T200 geometry (bob diameter 10.002 mm, cup diameter 10.845 mm). Each protein solution was placed on top of 500 μl of perfluorinated fluid (F-fluid) (Galden Perfluorinated fluid HT 70, Solvay Specialty Polymers) to decrease the sample volume. A solvent trap filled with tetradecane oil (Sigma-Aldrich) was used to avoid evaporation. Gel formation was followed by measuring changes in storage modulus as a function of time (sinusoidal deformation, frequency *f* = 1 Hz, strain *γ* = 1%). After 15 hours, the gel network structure was broken by carrying out a strain sweep (*f* = 1 Hz and *γ* = 0.1 to 100%, increasing at 10% steps) and the self-healing behavior was monitored by measuring the storage moduli as a function of time under sinusoidal deformation as mentioned above (*γ* = 1%). A maximum strain value of 100% was sufficient to break the gel completely. Additionally, after 15 hours of initial gelation, sample 100B 2% was broken (*f* = 1 Hz and *γ* = 0.01 to 1000%, increasing at 10% steps) repeatedly (5 times with 5.5 hour intervals), and the subsequent self-healing was recorded.

### Cell encapsulation

Cryopreserved MG-63 cells (ATCC^®^ CRL-1427^™^; LGC Standards GmbH), passage 36, were cultured for five days in proliferation medium (α-MEM 22571, Sigma-Aldrich) with 10% (v/v) FBS (Life Technologies), at 37°C, 95% relative humidity and 5% CO_2_. Prior to encapsulation, cells were washed twice with PBS, detached using trypsin-ethylenediaminetetraacetic acid (trypsin-EDTA; 0.25% (w/v) trypsin, 0.02% (w/v) EDTA) for 5 min, and resuspended at 10^7^ cells/ml in proliferation medium.

To obtain 0B, 50B, and 100B samples at 1%, 2% and 4% protein concentration a slightly modified procedure of that described in section 2.2 was used. First, appropriate protein solutions were prepared in 10 mM HCl. Next, 250 μl of each solution (2 samples for each protein composition: 0B, 50B, and 100B) was transferred to plastic vials, and 10 x PBS, HEPES buffer, and 0.1 M NaOH were added to adjust the protein concentrations and pH. After a pre-incubation period (duration specified in Table 2), during which the gels were allowed to reach a storage modulus of ~30–50 Pa, as determined by rheology on separate samples, 50 μl of cell suspension in proliferation medium was added (final cell density: 10^6^ /ml) and mixed gently. The initial gel formation step that preceded the cell seeding was included to minimize cell sedimentation inside the scaffolds. The relative volumes of protein-, buffer-, salt-, and cell-containing solutions used to prepare the various gel samples are shown in Table 3. After cell encapsulation, the protein solutions (50 μl aliquots) were immediately transferred to ThinCert™ Cell Culture Inserts for 24 well-plates (Item No.: 662641, Greiner Bio-One). Osteogenic medium (α-MEM supplemented with 10% (v/v) FBS, 100 U/ml penicillin, 10 μg/ml streptomycin, 50 mg/l ascorbic acid (Sigma), 10 nM dexamethasone (Sigma), and 10 mM β-glycerophosphate disodium salt hydrate (Sigma)) was added into the space surrounding the insert (1.2 ml/well) and scaffolds were incubated for 30 min at 37°C to allow setting of the gels. Afterwards, 200 μl of osteogenic medium was added on the top of each gel inside the ThinCert insert.

**Table 2:** Duration of initial gel formation step needed to reach a storage modulus of ~ 30–50 Pa.

Total protein concentration in scaffold (w/v)	Protein composition–concentration (w/v)		
	0B	50B	100B
1%	15–30 min	25–35 min	30–45 min
2%	4–8 min	8–15 min	10–17 min
4%	immediately	immediately	immediately

**Table 3:** Composition of cell culture scaffolds.

Total protein concentration in scaffold (w/v)	Protein in 10 mM HCl [μl]	10 x PBS [μl]	0.1M NaOH [μl]	HEPES [μl] (molarity)	Medium with cells [μl]
1%	250	50	50	100 (125mM)	50
2%	250	50	75	75 (166.6mM)	50
4%	250	50	137.5	12.5 (1M)	50

Additionally, two different types of cell-laden gels were prepared: (1) 50B 2% NB and (2) 50B 2% WP prepared directly in well-plates instead of inserts. For the 50B 2% WP sample, gel volume was ensured to be 48 μl, and 192 μl of osteogenic medium was added on top of each gel after 15 minutes of setting at 37°C. The volumes were chosen such that both the contact area: gel volume ratio and the contact area: medium (on top of the gel) volume ratio were kept constant for both the insert and the well plate.

As controls for the cell activity assay (see section 2.6), scaffolds without cells were incubated in osteogenic medium. All samples were prepared in triplicate (n = 3). The entire study was carried out for 7 days in an incubator at 37°C, 95% relative humidity and 5% CO_2_. The culture medium, for all the samples, was refreshed twice a week.

### Cell activity

Cell metabolic activity was measured with an alamarBlue^®^ (Invitrogen) assay for all scaffold types on 1, 3, and 7 days after cell seeding. The cell culture medium was exchanged for fresh medium with 10% (v/v) of alamarBlue^®^ reagent and incubated for 2–4 h (4 h on day 1, 3.5 h on day 3, and 2 h on day 7) at 37°C. Afterwards, medium containing the reagent was removed to measure fluorescence (ex 560 nm, em 590 nm; FLx800 reader, Bio-Tek Instruments). For scaffolds prepared in ThinCerts^™^, only medium from inside the insert was used for analysis. To compare cell activity on different scaffolds, the average fluorescence of the medium was calculated (n = 3), and the average signal from the corresponding scaffolds without cells was subtracted.

### Confocal microscopy and quantitative cell measurements

#### Confocal images

Fluorescent staining of F-actin and nuclei was performed as follows: The cell culture medium was removed from the inserts and from the well-plates. Scaffolds were washed with PBS and fixed for 2–4 h by addition of 0.3 ml of 3.3% paraformaldehyde (PFA) in PBS on top of the gels (into the inserts or in the case of 50B 2% WP scaffolds directly into the well), and stored in 70% (v/v) ethanol until imaging with a confocal microscope (Olympus FV1000 CLSM). Prior to imaging, the ethanol was removed, gels were washed twice in PBS, 300 μl permeabilization solution (1% Triton X-100 in PBS containing 1% FBS) was added on top of the scaffolds, and the samples were incubated for 20–30 min. The permeabilization solution was exchanged for 100 μl of staining solution consisting of Alexa Fluor® 568 conjugated to phalloidin (Life Technologies) for F-actin staining and DAPI (Life Technologies) for visualization of nuclei. Reagents were diluted in PBS containing 1% FBS, at 1:200 and 1:2500 ratio, respectively. After 1–2 hours of staining, gels were washed in PBS for 10 min, removed from the inserts or well-plates, and placed on glass coverslips for visualization.

For each sample (n = 3) of every gel type, three images were taken (in total n = 9), using an Olympus FV1000 CLSM with a 20 x water immersion objective, at random positions and varying depth of the focal plane, with the following exceptions: (1) Only two gels of 50B 1% and 100B 2% were visualized on day 1, and only two gels of 0B 1% on day 3; (2) owing to a strong red background from the scaffold protein, only DAPI staining was used for all 50B 2% samples on day 1, and for one sample of 50B 1% on day 1. Based on the obtained images, cell number, cell distribution, roundness index, and *R*_*A/N*_ ratio (stained F-actin area to stained nuclei area) were estimated.

#### Confocal image processing

Confocal images were processed with ImageJ 1.49o software. Every image was split into 2 channels (red for F-actin, blue for nuclei) and for each channel the background was automatically subtracted. Afterwards, each channel was converted to a black and white mask, based on an automatic threshold and manually adjusted when the contrast of the original image between cells and background was too low.

#### Cell number and cell distribution

To count the number of cells and to analyze the cell distribution, only the mask from the DAPI staining visualization was used, and a watershed filter was applied to separate the clustered nuclei. The ‘analyze particles’ function (settings: size of particles 40 –∞, circularity 0.25–1.00) was used to count the number of nuclei and therefore the number of cells. The ‘distribution analysis’ function was used to assess the cell distribution in space. This function gives values of the measured (*r*_*A*_) and theoretical (*r*_*E*_) average nearest neighbour distance, in which *r*_*E*_ is the expected mean nearest neighbor distance in the situation that all particles detected in the image are randomly distributed. The distribution index (*R*) was calculated [26]:

$$R=\frac{r_{A}}{r_{E}}$$ (2)

The equation gives values below 1 for clustered distributions, 1 for a random distribution, above 1 for more ordered patterns, and reaches a maximum for particles arranged in a perfect hexagonal lattice [26].

#### Cell roundness

To calculate the cell roundness index, the mask from F-actin staining was used with the ‘analyze particles’ function (settings: size of particles 40 –∞, circularity 0.0–1.0).

In ImageJ 1.49o software, roundness is calculated according to the formula:

$$Roundness\;index=\frac{4[Area]}{\pi {\left[Major\;axis\right]}^{2}}$$ (3)

The equation gives a maximal value of 1 for a perfect circle. A roundness index close to 1 indicates that a cell has few cellular extensions, whereas an index value closer to 0 corresponds to significant spreading of the cell.

#### R_A/N_ ratio of stained F-actin area to stained nuclei area

The *R*_*A/N*_ ratio was calculated by dividing the total area of stained F-actin by the total area of stained nuclei on each image. Area values were obtained from two separate masks (F-actin and DAPI staining) by using the ‘analyze particles’ function, with settings described above. Since decrease in nuclear-to-cytoplasmic ratio is a generally accepted indicator of cell maturation, spreading, or increase in cellular volume, and based on the fact that the cytoskeleton consists mostly of actin and tubulin networks, a higher value of *R*_*A/N*_ is considered as a sign of higher relative cytoskeleton area per cell, and consequently, more cellular extensions [27,28,29].

### Statistical analysis

Results were reported as the mean ± standard deviation. Statistical analysis was performed using SPSS Statistics software. Statistical differences were reported based on one-way Analysis of Variance (ANOVA) followed by a Least Significant Difference (LSD) post-hoc test for groups with homogeneity of variance, and by Games-Howell post-test for groups with heterogeneity of variance. Differences were regarded significant for p < 0.05.

## Results

In order to prepare a flexible and easily tunable fibrous hydrogel, we developed a modular self-assembling protein-polymer system. In this study both the silk-inspired protein-based triblock copolymer, **C**_**2**_**S**^**H**^_**48**_**C**_**2**_ and its cell-adhesive variant **B**^**RGD**^**C**_**2**_**S**^**H**^_**48**_**C**_**2**_ are used. These polymers are produced at high yield, up to 3.5 g/l of cell-free broth, as secreted proteins in the yeast *Pichia pastoris* [23,24].

### Porosity and adhesive motif density

Throughout the study, gels with varying porosity, in terms of the size of voids between fibers, and varying adhesive motif densities, were used. Since both parameters are crucial for the cell’s ability to spread and move in 3D, we determined their values, theoretically and/or experimentally, for samples with different total protein concentrations (1%, 2%, 4% (w/v)) and different RGD domain contents (0B, 50B, 100B). The characteristics of the tested samples are presented in Table 1 (see Materials and Methods).

Calculations of the size of the voids between fibers were performed assuming (1) a homogeneous fiber distribution throughout the gel, (2) a hypothetical cubic fiber arrangement in the gel, (3) an effective fiber thickness of *D*_*f*_ ~ 32 nm based on a fiber cross-sectional surface of *A*_*f*_ ~ 1018 nm^2^ (assuming a square fiber cross-section, by way of simplification), and (4) a protein density in the fibers of ~1.7 mmol/l [30]. Based on these assumptions, the linear size *l* of voids between two parallel fibers for 1, 2, and 4% **C**_**2**_**S**^**H**^_**48**_**C**_**2**_ gels is estimated as ~143, ~87, and ~48 nm, respectively, by solving Eq 4:

$${\left(l+D_{f}\right)}^{3}-(3(l+D_{f})A_{f}-2D_{f}^{3})\frac{\rho }{c}=0$$ (4)

where:

*l*–linear void size between two parallel fibers,

*A*_*f*_−fiber cross-sectional surface,

*D*_*f*_−effective fiber thickness,

*c*–protein concentration in mmol/l,

*ρ–*protein density in the fibers, equal to 1.7 mmol/l.

The derivation of Eq 4 is given in the S1 File.

Since the **B**^**RGD**^ extension in **B**^**RGD**^**C**_**2**_**S**^**H**^_**48**_**C**_**2**_ molecules represents only 3% of the total sequence length (Fig 1), the difference in fiber and void dimensions in comparison with gels made of unmodified **C**_**2**_**S**^**H**^_**48**_**C**_**2**_ is negligible. Thus, for 0B, 50B, and 100B gels with an equal total protein concentration, the size of the voids between the fibers is further assumed to be the same.

**Fig 2:**
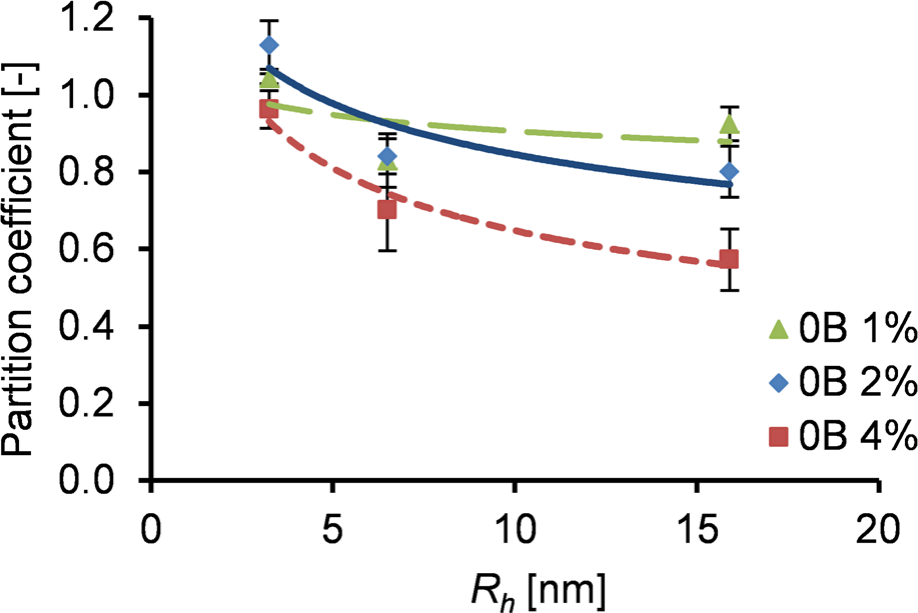
Partition coefficient of FITC-labelled dextran particles of different hydrodynamic radius in 0B gels of varying protein concentrations.

In addition, an approximate size range of the voids in the gels was estimated experimentally for different protein concentrations. Based on a diffusion study, we calculated the partition coefficient, defined as the ratio of dextran particles detected in the gel to the particles detected in the liquid (Fig 2). The partition coefficient is close to 1 if the particle concentration is equal throughout the system (both in the gel and liquid), indicating a fully permeable material. A lower partition coefficient indicates a less permeable material. All tested gels (1%, 2% and 4% (w/v)) were fully permeable for the smallest dextran particles (hydrodynamic radius of 3.25 nm). The largest particles (hydrodynamic radius of 15.9 nm) were not able to fully penetrate the gels, where the penetration reduction was less for gels with lower protein concentrations. These results are in reasonable agreement with our theoretical calculations and previous experimental work [31], confirming that the voids between fibers are in the nanometer range, with a decreasing size for increasing protein concentration.

Binding domain densities in the gels (50B and 100B) were calculated from the bulk protein concentration, noting that each **B**^**RGD**^**C**_**2**_**S**^**H**^_**48**_**C**_**2**_ molecule carries two RGD motifs. This implies for the 100B material an average bulk RGD domain density of 294 nmol/cm^3^ for 1% gels, twice this value for 2% gels, and a four-fold higher value for 4% gels. For 50B gels, the bulk density of RGD motifs is half the values calculated for 100B.

### Rheological characterization of hydrogels

**Fig 3:**
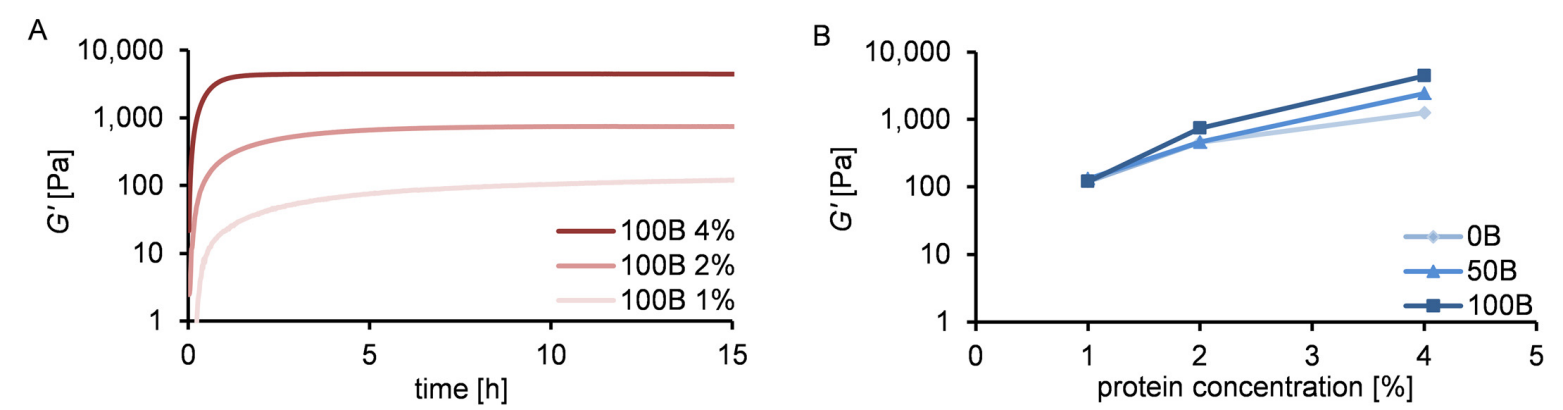
Rheological properties of hydrogels with different ratios of C_2_S^H^_48_C_2_ and B^RGD^C_2_S^H^_48_C_2_, and different final protein concentrations. (A) Time sweep for 100B material at 1%, 2% and 4% (w/v) of protein. (B) Final storage modulus dependency on protein concentration for gels with varying RGD domain density.

We characterized the rheological behavior of 1%, 2% and 4% hydrogels at physiological pH, in the presence of PBS and HEPES buffers. For all samples gel formation was observed; the storage modulus (*G’*) quickly dominated the loss modulus (*G”*). The final storage modulus *G’* increased strongly with increasing protein concentration (~110 Pa for 100B 1%, ~750 Pa for 100B 2%, ~4500 Pa for 100B 4%; Fig 3A). This can be attributed to an increased number of cross-links at higher protein concentrations. Fig 3B shows that the RGD density did not significantly affect *G’* of the formed gel. The observed variations in *G’* between 0B, 50B, and 100B gels at 2% and 4% can possibly be attributed to batch-to-batch variations of the protein materials [24]. We conclude that *G’* is tuned by the total protein concentration, whereas the RGD density is determined by the ratio between non-functionalized and functionalized protein polymers in the system. The final storage moduli and functional domain densities of the gels can thus be tuned independently.

**Fig 4:**
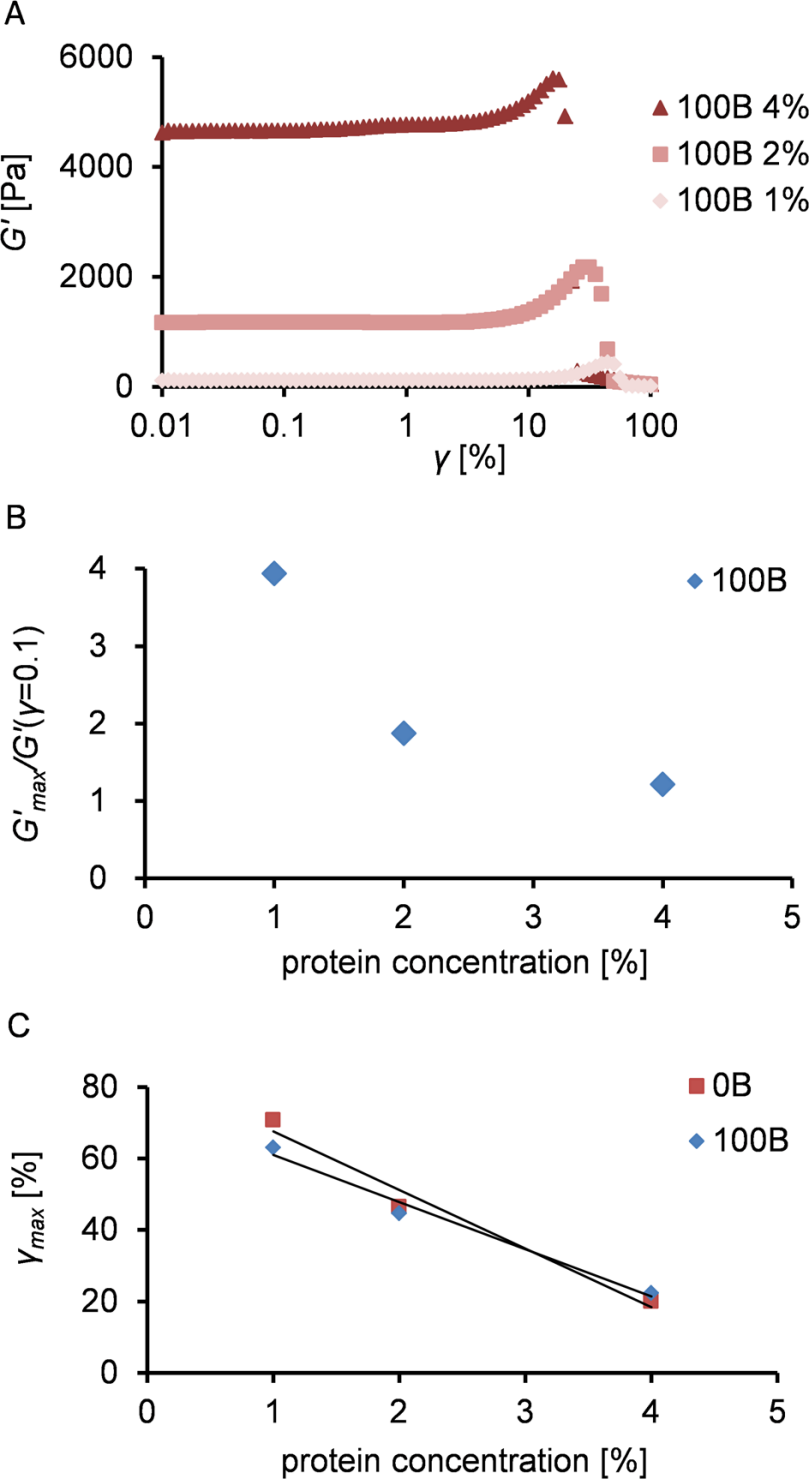
Strain sweep characteristics of C_2_S^H^_48_C_2_ and B^RGD^C_2_S^H^_48_C_2_ gels. (A) Storage modulus as a function of strain (*γ*) at various protein concentrations. (B) Maximal increase in storage modulus (strain stiffening) before gel rupture, in comparison with *G’*(*γ* = 0.1), for different concentrations of 100B material. (C) Strain at which gel ruptures (*γ*_*max*_) as a function of protein concentration for 0B and 100B material.

We subsequently analyzed the large deformation and rupture characteristics of the gels. Fig 4 shows the storage modulus of 100B as a function of strain, at different protein concentrations. All 100B samples revealed a clear strain stiffening effect: gel rupture was preceded by an increase in storage modulus (Fig 4A). The relative strain stiffening effect (defined as the ratio of maximum *G’* measured to the *G’* measured at *γ* = 0.1) decreased (Fig 4B) at higher protein concentrations. The strain at which the gel was destructed is lower for higher protein concentrations, irrespective of the presence of RGD domains (Fig 4C).

**Fig 5:**
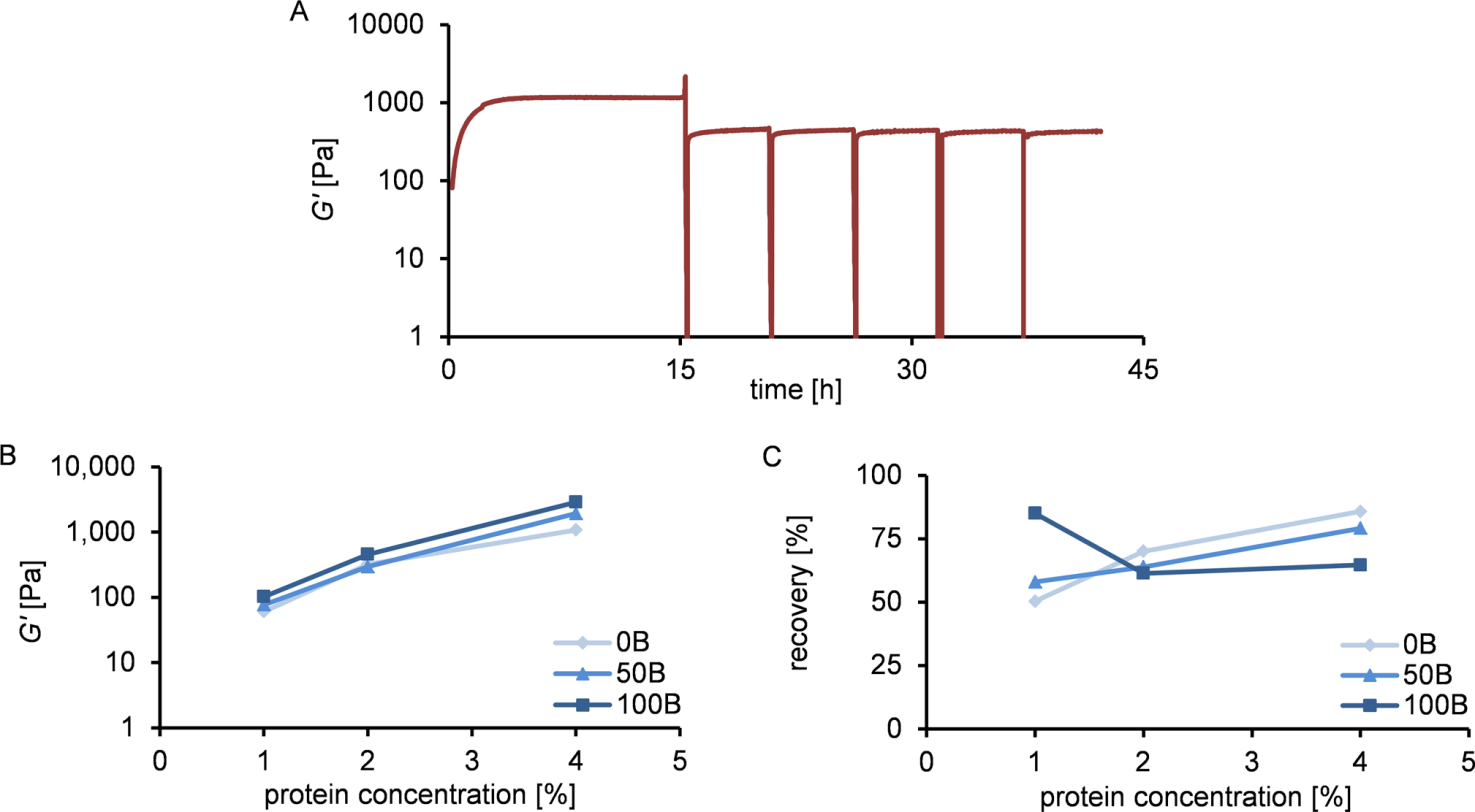
Ability to recover after breakage of hydrogels with different ratios of C_2_S^H^_48_C_2_ and B^RGD^C_2_S^H^_48_C_2_, and different final protein concentrations. (A) Time sweep for 100B 2% material, with several breaking cycles: gel formation and recovery after strain-induced breakage repeated 5 times in ~5.5 h intervals. (B) Storage modulus after one breakage and subsequent recovery as a function of protein concentration for gels with varying RGD domain density. (C) Percentage of storage modulus recovered after one breakage as a function of protein concentration for gels with varying RGD domain density.

Finally, the ability of gels to self-heal after rupture was explored. The response of the gels to repeated breaking is shown in Fig 5A, as illustrated by 100B 2%. We find that the material has the ability to rapidly and repeatedly recover to the same modulus after multiple rupture events. Recovery appeared to be much faster than the initial gel formation (compare with Fig 3A). While initial gelation requires the formation of new, slowly growing fibers, recovery is fast because it occurs through fiber rearrangements [19]. Irrespective of RGD density, all gels recovered to 50–85% of the initial *G’* value after the first rupture event (Fig 5B and 5C). We conclude that *G’* after breakage and subsequent recovery is determined by the total protein concentration only, similarly as observed for freshly prepared gels. The extent of recovery decreased with decreasing protein concentration, except for the 100B 1% sample. The higher extent of recovery for more concentrated gels can probably be attributed to the closer proximity of fibers and easier formation of new cross-links [19].

Since it was shown before that higher ionic strength may decrease stiffness and gelation speed of **C**_**2**_**S**^**H**^_**48**_**C**_**2**_ [19], we tested the 50B 2% NB sample prepared with a lower salt concentration (used in the cell culture study to analyze the possible influence of HEPES and ionic strength on cell behavior), and compared it with 50B 2% (see S1 Fig). No significant differences in storage modulus or the kinetics of gel formation were observed.

### Cell culture

Cell culture scaffolds were prepared in ThinCert^™^ Cell Culture Inserts. The chosen insert type was one with a high pore density to ensure good accessibility of nutrients and oxygen from the surrounding medium. We encapsulated MG-63 cells in the hydrogels and analyzed the cellular response to different protein concentrations and functional motif densities during the 7 days of culture. Different preparation methods were used for the control samples, which will be discussed later in this section.

**Fig 6:**
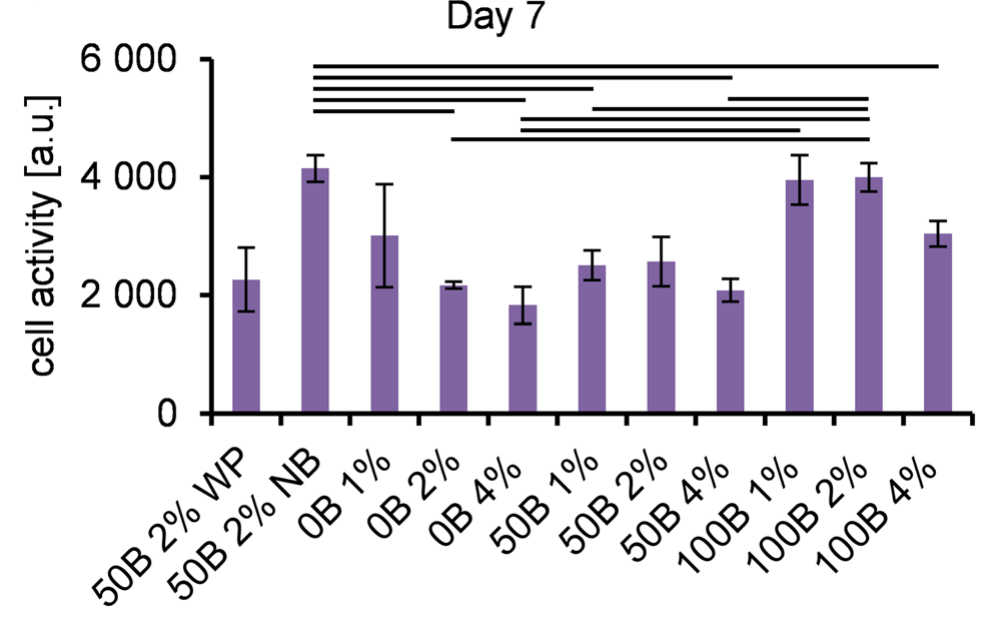
Cell metabolic activity. Determined by the alamarBlue^®^ assay on day 1.

To estimate the viability and proliferation of encapsulated cells, the metabolic activity was tested with the alamarBlue® assay. Fig 6 and S2 Fig show that all scaffolds were able to support viable cells for 1, 3, and 7 days of 3D culture. At day 1 (S2 Fig), we observed a large variation in results for individual scaffold types, most likely indicating that the cells needed time to adapt. After 7 days of cell culture (Fig 6), cells with the highest metabolic activity were observed in 100B 1% and 100B 2% samples, indicating that a high adhesive motif density together with a low protein concentration (low matrix stiffness) is most beneficial for cell viability.

**Fig 7:**
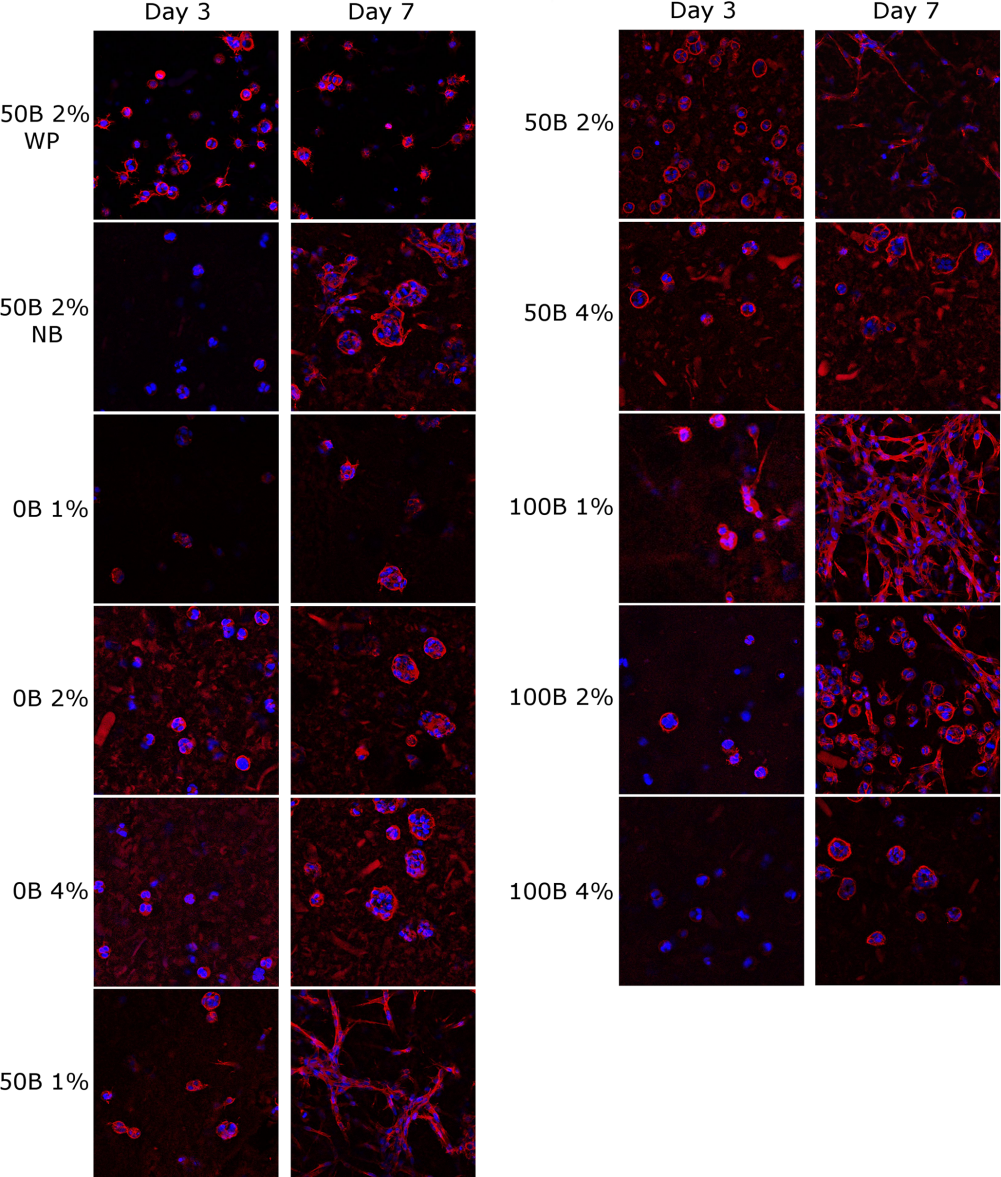
Morphology of fluorescently stained MG-63 cells encapsulated in silk-inspired protein scaffolds, with different total protein concentration and RGD domain density at day 3 and 7 of culturing. Red: actin; blue: nuclei. Image size: 370 × 370 μm.

To further analyze cell spreading and cytoskeletal development, confocal images of nuclei (DAPI) and F-actin (phalloidin) were made on day 1, 3, and 7, followed by quantitative analysis. Fig 7 shows representative images for comparison of cell behavior in different scaffolds on day 3 and 7. In the absence of RGD domains, no cell spreading was observed, regardless of the total protein concentration. In contrast, for cells encapsulated in RGD-containing gels (both 50B and 100B) some cellular extensions were visible already on day 3 and to a greater extent on day 7, but not for scaffolds with 4% (w/v) protein concentration, where cells remained round. The most spread morphology, with a seemingly interconnected cellular meshwork, was adopted by the cells embedded in the functionalized, low-modulus 100B 1% and 50B 1% scaffolds. This indicates that high gel stiffness can have an inhibitory effect.

**Fig 8:**
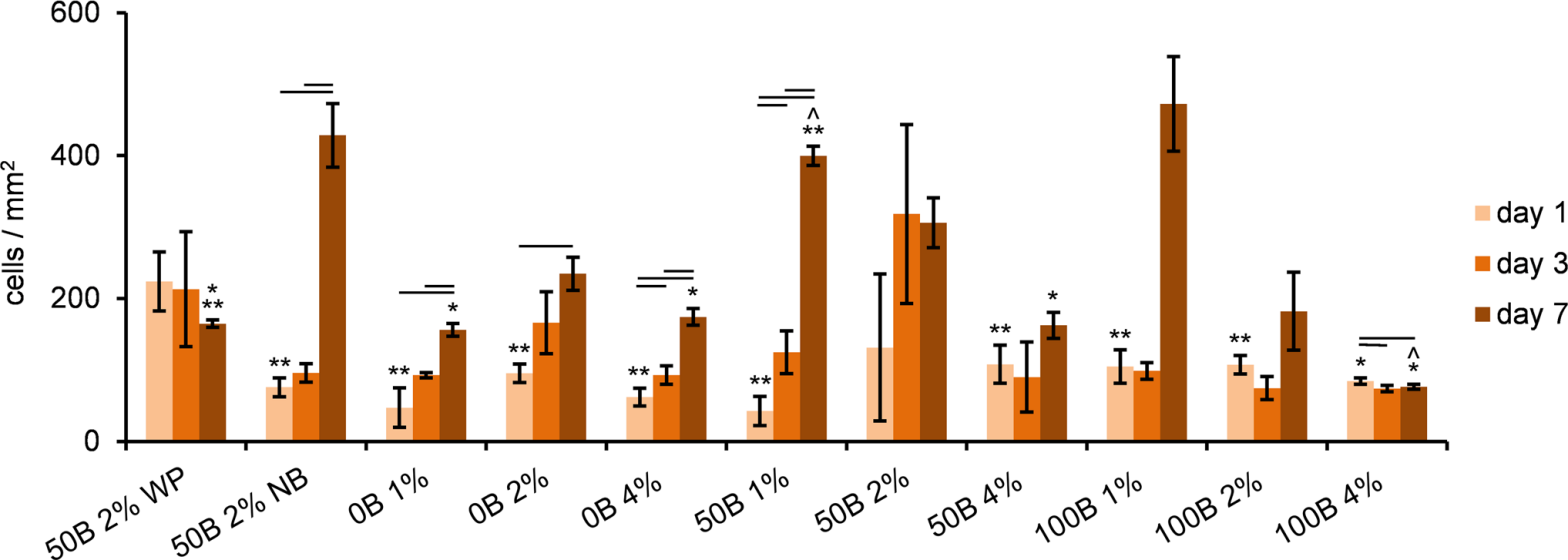
Quantitative determination of MG-63 cell number based on analysis of confocal images. Significant differences between different days of cell culture for the same scaffold type are marked with horizontal lines; ^ significant difference relative to 50B 2% WP scaffold; * significant difference relative to 50B 1%; ** significant difference relative to 100B 4%; p < 0.05.

Cell counting based on confocal images revealed a significant increase in cell number over time (i.e. proliferation) in all 0B scaffolds and in 50B 1% (Fig 8). Owing to the larger variance in the total cell number in the gels containing RGD domains, no significant changes were found for the other 50B and 100B scaffolds. On day 7, the highest cell number was detected for 50B 1% and 100B 1%, while the lowest cell number was detected for the 100B 4% scaffold, indicating a negative influence of higher protein concentration on proliferation.

**Fig 9:**
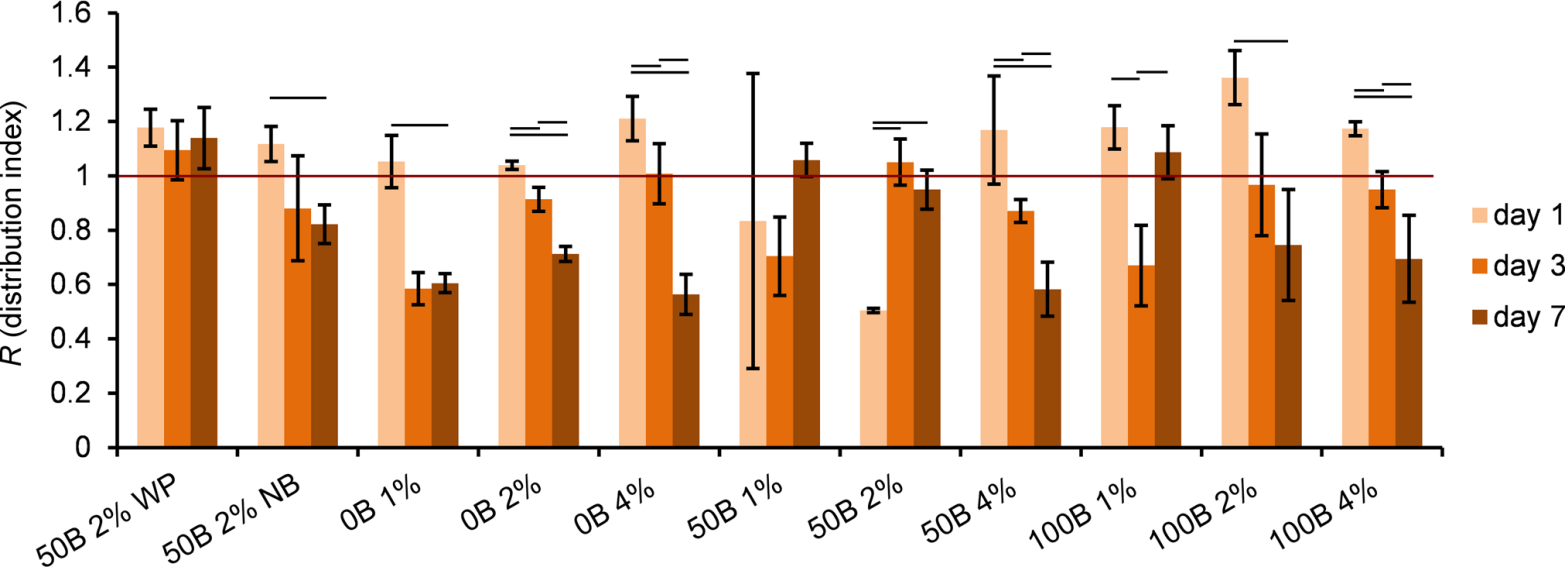
Determination of MG-63 cell distribution inside scaffolds, based on analysis of confocal pictures. Significant differences between different days of cell culture for the same scaffold type are marked with horizontal lines; p < 0.05. Values below 1 indicate clustered distributions, equal to 1 –a random distribution, and above 1 –more ordered patterns.

Based on the cell distribution analysis (Fig 9), a tendency to form clusters over time was observed for scaffolds without RGD domains and scaffolds with the highest protein content. At the end of the cell culture experiment (day 7), cells were most evenly distributed in 50B 1%, 50B 2%, and 100B 1%. Low protein concentrations and the presence of RGD appeared to be beneficial not only for cell proliferation, but also for cell migration and motility.

**Fig 10:**
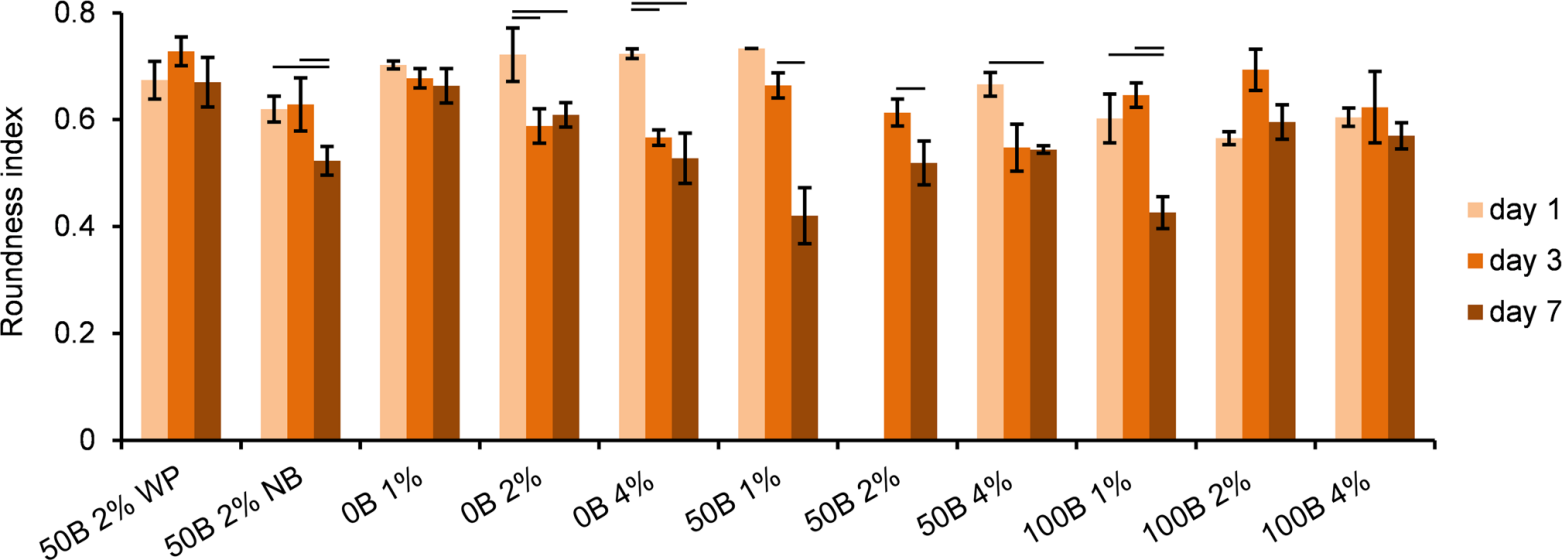
Quantitative determination of MG-63 cell roundness, based on analysis of confocal images. Significant differences between different days of cell culture for the same scaffold type are marked with horizontal lines; p < 0.05. The data analysis is focused on relative changes over time rather than on absolute values. Owing to a strong red background from the scaffold protein no data could be obtained for 50B 2% samples on day 1.

Alexa Fluor^®^ 568 conjugated to phalloidin appeared not to selectively stain F-actin, but it also stained the scaffold. As a consequence, more background signal was visible in red for higher protein concentrations, which influenced the appearance of the mask computed from the F-actin staining channel, and therefore, the outcome of the roundness and *R*_*A/N*_ calculations as well. Thus, we focused the data analysis on relative changes over time rather than on absolute values. Quantitative analysis of cell roundness (Fig 10) indicated that cell spreading was enhanced over time in most of the gels, with the strongest effect for 50B 1% and 100B 1% scaffolds. Additionally, calculations of *R*_*A/N*_ ratio (see S3 Fig) revealed that the most pronounced development of cytoskeletal structure in time appeared in 100B 1% and 100B 2% scaffolds.

In addition, we included two control samples: 50B 2% WP and 50B 2% NB. Differences between 50B 2% WP and 50B 2% samples were not detected at any measured time point in cell activity (Fig 6 and S2 Fig). For 50B 2%, more clustering than for 50B 2% WP at day 7 (Fig 9), and a small decrease in roundness was observed (Fig 10). In general, no strong effects were noticed indicating that the accessibility of nutrients and oxygen is not a limiting factor in this study. The cell culture results for cells encapsulated in 50B 2% NB as compared to 50B 2% scaffolds did not reveal pronounced differences either. Only on day 1 of the culture, cells were significantly more clustered when encapsulated in 50B 2%; however, this effect disappeared in time. In conclusion, there was generally no influence of the HEPES or PBS buffer on cell behavior at the concentrations used in the study.

## Discussion

Based on the growing need to develop 3D cell culture systems, which more accurately mimic the environment of natural tissues and cell–matrix interactions, we evaluated the performance of our genetically engineered silk-inspired material system [19,32] with respect to cell adhesion and growth in 3D, by independent variation of the RGD motif density and the total protein concentration.

The cell metabolic activity test and the analysis of confocal images, both qualitatively and quantitatively, clearly showed a positive effect of RGD motifs (at lower matrix stiffness) on cell viability, growth, and morphology. In general, cells encapsulated in 50B and 100B materials performed better than those in 0B. We conclude that for improved cell behavior, RGD functionality is a necessary, though insufficient, requirement. The gel matrix also needs to exhibit the proper mechanical properties (e.g., compliance) and/or architecture (e.g., fibrous structure or presence of proteolytically sensitive sites) for cells to grow and function properly.

To estimate the influence of matrix rheological properties on cellular response, we tested 1%, 2% and 4% (w/v) gels. The storage moduli of the fully set gels were strongly affected by the total protein concentration, increasing by two orders of magnitude, when going from a concentration of 1% to 4%. For all tested samples, the ability of the network to self-heal was observed. According to the results of the cell culture study, cells were able to spread in the scaffolds with lower protein concentrations (i.e., 1% and 2%), but were hampered in the case of 4% protein concentration. This indicates that more concentrated gels, even in the presence of cell adhesion motifs, probably cause some sort of mechanical obstruction, which is in agreement with other studies [33,34]. In densely cross-linked or entangled gels, lacking engineered degradation sensitive sites, it is most probably more difficult for cells to move fibers and deform the network or to break the higher number of physical cross-links. Therefore, we conclude that, besides the presence of RGD domains, gels have to be compliant to allow deformation of the matrix by moving or spreading cells. The relevant mechanical property here is probably the yield stress rather than the storage modulus, but these two phenomena are highly correlated. The exact molecular mechanism that determines the ability of cells to spread by changing the gel stiffness cannot be elucidated in this study. Nevertheless, the obtained results are in agreement with other reports showing that more compliant hydrogel scaffolds enhance cell spreading [33], especially in combination with the presence of RGD domains [1,8,16,35]. Hence, essential requirements for proper spreading of encapsulated cells include a matrix design allowing cell migration and growth (in our study ensured by gel compliance) as well as sufficient cell-adhesion sites.

Another possibility of designing a matrix favorable for cells would be the incorporation of degradation sensitive domains (e.g., MMPs sensitive sites) or introduction of microporosity. For the gels presented in this study, the voids are far too small to allow unrestricted cell motion. The size of the voids, as estimated assuming a homogeneous network and determined by an experimental diffusion study, is in the range of 30–150 nm and decreases with increasing protein concentration. Hence, the voids are always orders of magnitude smaller than the dimensions of cells, and as such here do not determine the extent of spreading. When not opting for the possibility of introducing micropores e.g. via the use of porogens, pore size is thus not a relevant parameter for tuning in this hydrogel system, unlike gel stiffness.

Our gels revealed several features mimicking the natural ECM: (1) The pore size is in the same range as the pore sizes of natural ECM (5–400 nm) [2]; (2) Similarly, the fiber thickness (~32 nm diameter) resembles that of ECM fibrous components (fibronectin: 2–3 nm diameter, collagen: 50–200 nm diameter) [2]; (3) Also, the non-linear rheological behavior of our gels resembles that of collagen type I networks [36], and other biopolymer gels [37]. Typically for natural networks, the degree of strain-stiffening and maximal deformation before breaking are higher when the protein concentration in the gel is lower, which is in agreement with our data; (4) The ability of the gels to largely recover after the first breakage in the rheometer, and to repeatedly recover to the same modulus after subsequent, multiple breaking cycles, has also been observed for collagen [36]. The limited drop in storage modulus that occurs after the first breakage and recovery of the gel (50–85%), can be assigned to irreversible damage of some of the fibers. The subsequent reproducible recovery indicates that, in addition to this irreversible damage, reversible reorganization of the fibers is taking place, most probably due to the presence of weak physical cross-links between the fibers [19,31]. This suggests that the material could potentially respond to cell-induced stress (provided that it exceeds the local yield stress) [38] by breaking and subsequent reforming of the gel, while maintaining integrity over time. In natural ECM, the strain-stiffening, as well as the ability to self-heal, serves to keep the integrity of the network [36,39]. The stiffening mechanism, together with fiber rearrangements and changes in the matrix geometry, is also most probably a way of long-distance cell–cell communication, allowing sensing of neighboring cells through alterations in network contraction [39,40]. Moreover, mechanical changes of the matrix can affect cell motility and proliferation, since many cell types migrate towards increased stiffness [41].

To the best of our knowledge the hydrogel system presented in this study, is the first material that combines (1) mild conditions for cell encapsulation, (2) no need for additional cross-linking, (3) biomechanical features resembling ECM and (4) precise control over biochemical signals density. Material systems featuring one or several of these aspects have been described before [1,13,14,15,16,33,34,35], but not all in one system. We believe that this combination is important, because the independent control over mechanical and biological properties in our material design may allow for systematic and detailed studies on parameters influencing cell behavior in an ECM mimicking environment. For example, the influence of different active domians and their occurence at different densities in the material can be analyzed in relation to the mechanical interactions between cells and individual fibers in the material. Moreover, recently we showed that the fibers in this system can be bundled controllably, which is another interesting ECM mimicking parameter to study as a function of cell response.

Adhesion-dependent MG-63 cells, for which spreading is an essential factor [3] and which exhibit an integrin subunits profile similar to that of human primary osteoblasts [22], were used in this study. This cell line has been previously used in several studies in 3D collagen culture systems [42,43,44,45], revealing the ability to proliferate, migrate [43] and differentiate into osteoblast phenotype [46] inside the scaffolds. In contrast to collagen gels [46,47,48], our material did not contract or shrink in the presence of cells. The proposed hydrogel system can be further optimized for specific applications by introducing other biochemical cues. For example, the enhancement of osteoblastic cell performance, especially spreading, could be achieved by the incorporation of KRSR (Lys-Arg-Ser-Arg) proteoglycan binding domains. Design of these domains is based on a basic-basic-nonbasic-basic amino acid sequence found in bone adhesive proteins [49] and were shown to be selective adhesion sites for osteoblastic cells [50,51,52]. The effect of these domains on cell behavior in 3D cell culture, as well as the effect of incorporation of degradation-sensitive domains, such as matrix metalloproteases-sensitive sites, could be explored.

## Conclusions

We described the preparation of self-assembled fibrous hydrogel scaffolds for 3D cell culture from the genetically engineered silk-inspired polymer **C**_**2**_**S**^**H**^_**48**_**C**_**2**_ and its RGD-functionalized variant **B**^**RGD**^**C**_**2**_**S**^**H**^_**48**_**C**_**2**_. The RGD motif density in the gels was precisely controlled by mixing the two proteins in variable proportions, and tuned independently of matrix stiffness. We showed that for improved behavior of cells encapsulated in the gels, RGD functionality is not the sole requirement; space for cells in the gel matrix and/or the ability of cells to deform the fibrous network are crucial. The results indicate that our fibrous protein-polymer hydrogels are suitable to study cellular responses under highly defined ECM- mimicking conditions owing to the combination of controlled incorporation of (1) biochemical cues and (2) mechanical features together with (3) mild encapsulation conditions. In addition, the ability to quickly and repeatedly recover makes the material a very good candidate for injectable drug delivery or tissue regeneration systems [53], and can be advantageous for substrate handling, since in case of unintended fracture during in situ application, the network can be restored.

## Supporting Information

S1 Fig Time sweep for 50B 2% NB and 50B 2% hydrogel. Gel formation and recovery after strain-induced breakage of the gel at t ~15 h.
(TIF)

S2 Fig Cell metabolic activity. Determined by the alamarBlue^®^ assay: (A) on day 1, (B) on day 3. Significant differences between samples are marked with horizontal lines, p < 0.05.
(TIF)

S3 Fig Quantitative determination of MG-63 cell *R*_*A/N*_ ratio of stained F-actin area to stained nuclei area, based on confocal images analysis. Significant differences between different days of cell culture for the same scaffold type are marked with horizontal lines; p < 0.05. The data analysis is focused on relative changes over time rather than on absolute values.
(TIF)

S1 File The derivation of Eq 4. (PDF)
