# Correction: Fibrous Hydrogels for Cell Encapsulation: A Modular and Supramolecular Approach

**DOI:** 10.1371/journal.pone.0159893

**Published:** 2016-07-18

**Authors:** The PLOS ONE Staff

There is an error in the third sentence of the Abstract. The correct sentence is: Here we present an ECM-mimicking genetically engineered protein-based hydrogel as a 3D cell culture system that combines several key features: (1) Mild and straightforward encapsulation of the cells, without the need of an external crosslinker. The publisher apologizes for the error.
