# Supplementary figures and images for: Fibrous Hydrogels for Cell Encapsulation: A Modular and Supramolecular Approach
Source: PLoS One. 2016 May 25;11(5):e0155625. doi: 10.1371/journal.pone.0155625 (PMC4880210; doi:10.1371/journal.pone.0155625)

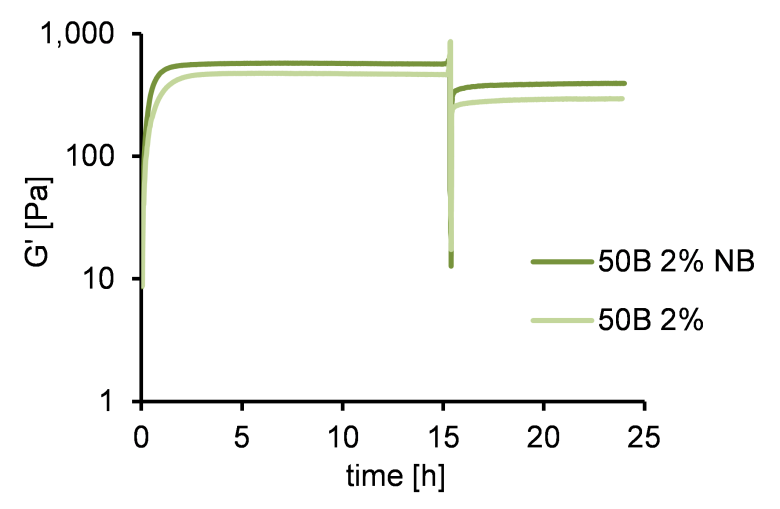

Supplement: S1 Fig — Gel formation and recovery after strain-induced breakage of the gel at t ~15 h. (TIF) [file pone.0155625.s001.tif]

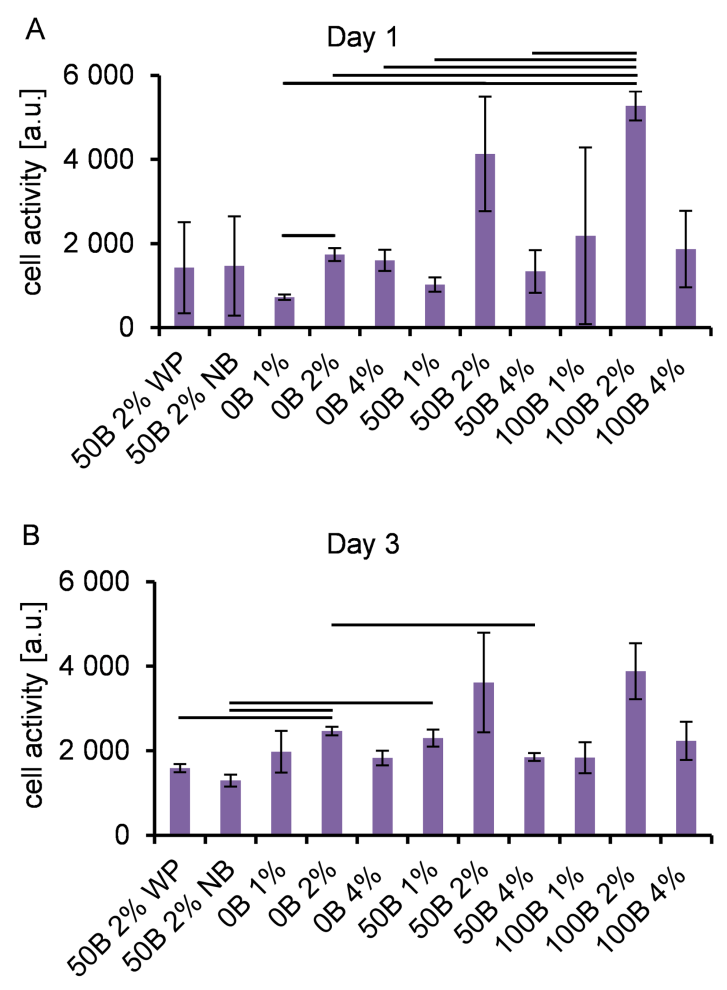

Supplement: S2 Fig — Determined by the alamarBlue® assay: (A) on day 1, (B) on day 3. Significant differences between samples are marked with horizontal lines, p < 0.05. (TIF) [file pone.0155625.s002.tif]

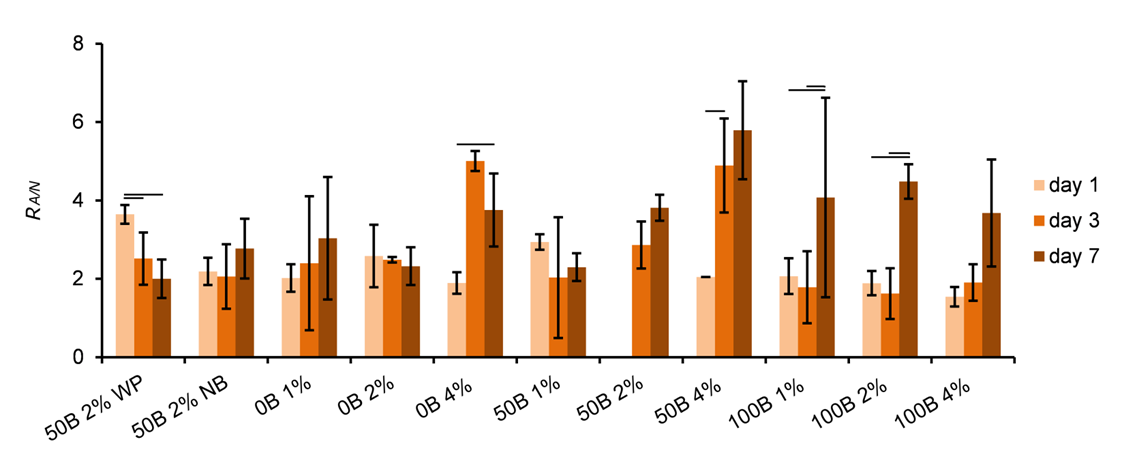

Supplement: S3 Fig — Significant differences between different days of cell culture for the same scaffold type are marked with horizontal lines; p < 0.05. The data analysis is focused on relative changes over time rather than on absolute values. (TIF) [file pone.0155625.s003.tif]
