# Supplementary material for: Fibrous Hydrogels for Cell Encapsulation: A Modular and Supramolecular Approach
Source: PLoS One. 2016 May 25;11(5):e0155625. doi: 10.1371/journal.pone.0155625 (PMC4880210; doi:10.1371/journal.pone.0155625)
Supplement: S1 File — (PDF) [file pone.0155625.s004.pdf]

The derivation of equation 4.

To calculate the void size between two parallel fibers, a homogeneous cubic arrangement of fibers is assumed, as depicted below:

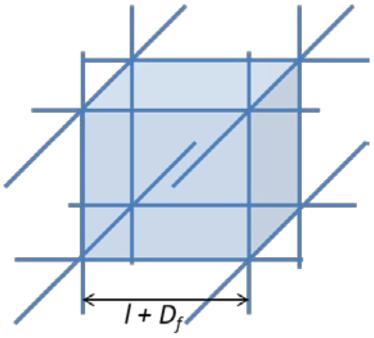

In which l is the linear void size between two parallel fibers and D_f the effective fiber thickness. The volume, V, of one cubic unit will therefore be: $V = \left(l + D_f\right)^3$

In addition, we assume a square fiber cross-section as depicted in the next schematic:

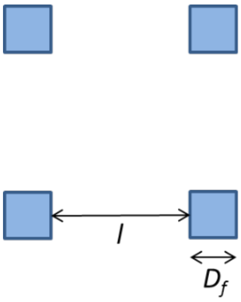

The fiber cross-sectional surface, A_f, is therefore: $A_f = D_f^2$
The volume of a fiber at one of the edges of the cubic unit is: $(l + D_f)A_f$
One cubic unit contains 12 of these fiber edges. Each edge is shared by 4 cubic units, so ¼ of each fiber contributes to the volume that is occupied by fiber within one cubic unit:

$$3(l + D_f)A_f$$

In addition, in this model, at each corner three fibers overlap, which should be corrected for. Therefore the volume within one cubic unit that is occupied by fiber, V_f, is:

$$V_f = 3(l + D_f)A_f - 2D_f^3$$

From a previous study it is known that the protein density, ρ, in the fibers is 1.7 mmol/l. Therefore the number of protein molecules present within one cubic unit is:

$$\rho V_f = \rho(3(l + D_f)A_f - 2D_f^3)$$

Finally, the volume of one cubic unit, V, is equal to the number of protein molecules per cubic unit divided by the protein concentration, c (in mmol/l).

$$V = \rho V_f / c$$

$$\rho(3(l + D_f)A_f - 2D_f^3)/c = \left(l + D_f\right)^3$$

This is equivalent to equation 4.
